# Potential Anti-Cancer Drug 6RK73 Suppresses Ovarian Cancer Growth by Inactivating the AKT1/Sp1 Induced c-Myc Signaling Pathway

**DOI:** 10.7150/jca.113511

**Published:** 2026-01-01

**Authors:** Sisi Kuang, Weifeng Feng, Siqi He, Wei Meng, ChunYan Yang, Yingxia Ning

**Affiliations:** 1 Department of Gynaecology and Obstetrics, The First Affiliated Hospital of Jinan University, Guangzhou, Guangdong, China.; 2 Department of Gynaecology and Obstetrics, The First Affiliated Hospital of Guangzhou Medical University, Guangzhou, Guangdong, China.; 3 Department of Reproductive Medicine, Shenzhen People's Hospital, The First Affiliated Hospital of Southern University of Science and Technology, Shenzhen, Guangdong, China.; 4 Department of Traditional Chinese Medicine, The First Affiliated Hospital of Jinan University, Guangzhou, Guangdong, China.; 5 Faculty of Arts and Science,University of Toronto, Toronto, Ontario, Canada.; 6 Department of Gynaecology and Obstetrics, The second people's hospital of Huaihua City (Tumor hospital of Huaihua City), Huaihua, Hunan, China.

**Keywords:** 6RK73, ovarian cancer, UCHL1, AKT1, Sp1, c-Myc

## Abstract

6RK73 is a novel drug designed to target UCHL1 deubiquitinase. Preliminary studies have indicated its anti-cancer activity in breast cancer and renal cell carcinoma. However, its potential anti-cancer effects in other malignancies, including ovarian cancer, remain unclear. In this study, we first determined the IC50 values of 6RK73 in ovarian cancer cell lines OVCAR3 and SKOV3, which were 10.62 μM and 12.90 μM, respectively. Subsequently, we found that 6RK73 effectively inhibited cell proliferation and arrested cell cycle progression in ovarian cancer cells *in vitro*. Furthermore, 6RK73 suppressed the formation of subcutaneous ovarian cancer tumors in nude mice. Mechanistically, 6RK73 significantly inhibited the AKT1/Sp1/c-Myc signaling pathway, which not only disrupted the interaction between Sp1 and c-Myc but also reduced Sp1 deubiquitination, thereby downregulating c-Myc protein expression. Interestingly, the anti-tumor effects of 6RK73 in ovarian cancer were independent of UCHL1 inhibition. Finally, AKT1 overexpression reversed the 6RK73-mediated suppression of cell proliferation by reactivating the AKT1/Sp1/c-Myc signaling pathway. These findings suggest that 6RK73 is a promising anti-cancer agent that exerts its effects by inactivating AKT1/Sp1/c-Myc signaling in ovarian cancer.

## Introduction

**Figure 1:**
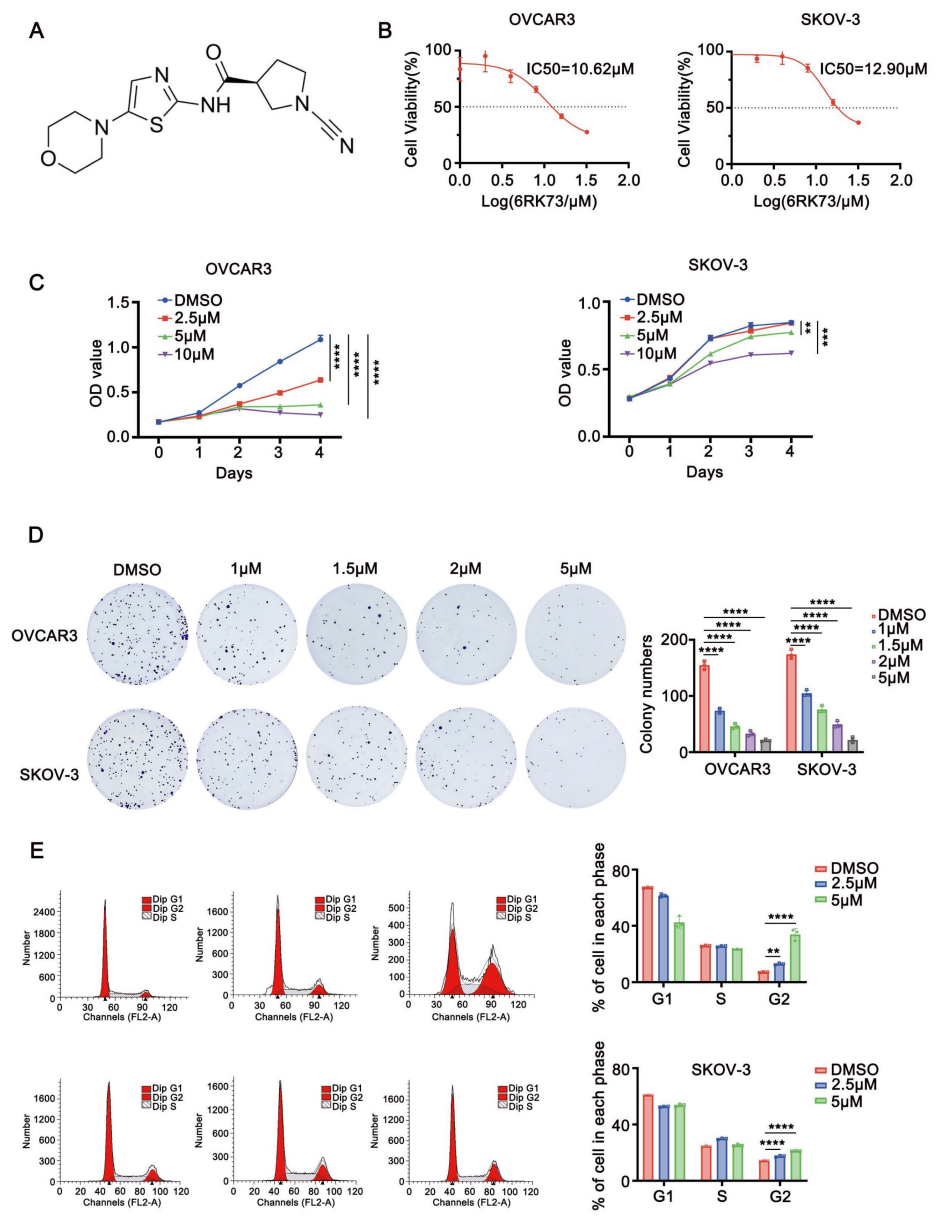
** Anti-ovarian cancer effects of 6RK73. (A-E)** A. Molecular structural formula of 6RK73; B. The IC50 of 6RK73 in OVCAR3 and SKOV-3 were 10.62 µM and 12.90 µM, respectively; C. 6RK73 inhibits cell proliferation in a dose-dependent manner (0, 2.5, 5, 10 µM); D. Colony formation assays in OVCAR3 and SKOV-3 cells treated with increasing concentrations of 6RK73 (0, 1, 1.5, 2, 5 µM); E. 6RK73 induces G2/M phase cell cycle arrest in a concentration-dependent manner (2.5 and 5µM) in OVCAR3 and SKOV-3 cells.

6RK73, with the molecular formula C₁₃H₁₇N₅O₂S (Figure 1A), is a covalent, irreversible, and specific inhibitor of UCHL1, a deubiquitinase that plays a dual role in tumor progression^1^-^4^. 6RK73 contains a unique cyanoimide functional group that enables covalent binding to thiol residues within the active site of UCHL1, resulting in the formation of isothiourea adducts and subsequent loss of enzymatic activity^5^, ^6^. Functionally, 6RK73 exhibits an inhibitory effect on UCHL1 comparable to genetic knockdown, while demonstrating high selectivity toward UCHL1 over related enzymes. Previous studies have reported that 6RK73 suppresses oncogenic UCHL1 activation in breast cancer and delays the progression of renal cell carcinoma (RCC) associated with Xp11.2 translocation/TFE3 gene fusion, particularly PRCC-TFE3 tRCC^7^. However, its anti-cancer efficacy in other malignancies, including ovarian cancer, remains unclear.

Ovarian cancer is the fifth leading cause of cancer-related mortality among women in the United States^8^. Globally, approximately 140,000 women succumb to this disease each year. The etiology of ovarian cancer is complex, and its highly malignant nature often results in a poor prognosis. Due to the absence of specific symptoms in the early stages, most ovarian cancer patients are diagnosed at an advanced stage, limiting their treatment options. Therefore, the development of novel therapeutic agents for ovarian cancer is urgently needed.

In this study, we observed that 6RK73, as a potential anti-cancer agent, significantly inhibited ovarian cancer cell growth and cell cycle progression by attenuating AKT1/ Sp1/c-Myc signaling, independent of UCHL1 inactivation.

## Material and Methods

### Cell culture and reagents

Ovarian cancer cell lines SKOV-3 and OVCAR3 were obtained from the Shanghai Institute of Cell Biology, Chinese Academy of Sciences (Shanghai, China). Cells were cultured in RPMI 1640 medium (Gibco) supplemented with 10% fetal bovine serum (FBS, HyClone) at 37 °C in a humidified incubator with 5% CO₂. The compound 6RK73 was purchased from Med Chem Express (USA).

### Cell counting kit 8 assay

The CCK-8 assay was used to assess cell proliferation and cytotoxicity. For proliferation assays, cells were transfected with UCHL1 for 36 h and subsequently seeded into 96-well plates at a density of 2,000 cells per well. Once the cells adhered, CCK-8 reagent was added to the culture medium at a 1:100 dilution. After incubating at 37 °C for 2 h, absorbance was measured at 450 nm. For cytotoxicity assays, cells were inoculated into 96-well plates (2,000 cells per well) and treated with varying concentrations of 6RK73 for 48 h. After adding the CCK-8 reagent, the cells were incubated for 2 h, and optical density (OD) values at 450 nm were recorded.

### Colony formation assay

Cells were seeded into six-well plates at a density of 200 cells per well. In the 6RK73-treated group, cells were exposed to a concentration gradient of 6RK73 (1 µM, 1.5 µM, 2.5 µM, and 5 µM) after adherence. Colonies were cultured for 12 days. After the incubation, colonies were fixed with 4% polyformaldehyde for 15 minutes, and stained with crystal violet for 15 minutes. Then, plates were rinsed with running water, photographed, and analyzed using ImageJ software.

### EdU incorporation assay

Cells were plated in 96-well plates at a density of 5,000 cells per well. The Cell-Light EdU Apollo 488/567 *In Vitro* Imaging Kit (RiboBio) was used to assess cell proliferation. After incubation with 10 μM EdU for 2 h at 37 °C, cells were fixed with 4% paraformaldehyde, permeabilized with 0.3% Triton X-100, and stained with Apollo fluorescent dyes. Nuclei were counterstained overnight with DAPI (Beyotime Biotechnology, Shanghai, China). The proportion of EdU-positive cells was quantified using ImageJ software.

### Cell cycle analysis

A total of 5 × 10⁶ ovarian cancer cells were collected after 48 h of incubation. Cells were resuspended in PBS and centrifuged at low speed. Following the manufacturer's instructions, 500 µL of staining solution was added to each sample, vortexed, and incubated at 37°C for 30 minutes. After an additional incubation at 4°C for 30 minutes, flow cytometry was performed.

### Cell transfection

Plasmids encoding UCHL1 (pcDNA3.1-UCHL1) and AKT1 (pENTER-AKT1) were obtained from GeneChem (Shanghai, China), while those encoding Sp1 (pENTER-Sp1) and USP7 (pLV3-USP7) were purchased from Vigene Biosciences (Jinan, China). Cells were seeded in six-well plates and incubated at 37°C for 12 hours. When cell confluency reached approximately 60% the following day, transfection was carried out using Lipofectamine 3000 (Invitrogen, Carlsbad, CA, USA) in accordance with the manufacturer's protocol. The culture medium was replaced 12 hours post-transfection, and cellular protein lysates were collected 48 hours later for subsequent analysis.

### Subcutaneous tumor model in nude mice

To evaluate the anti-tumor effects of 6RK73 *in vivo*, a subcutaneous tumor model was established in nude mice. Four-week-old nude mice were purchased from SPF (Beijing) Biotechnology Co. Ltd. (Beijing, China) and acclimated to an SPF-grade environment for one week. OVCAR3 cells (6×10⁶) were subcutaneously injected into the mice, which were then divided into two groups (n = 5): control and 6RK73 -treated groups. Starting on day 7 post-injection, mice in the 6RK73 group received intraperitoneal injections of 10 mg/kg 6RK73 every two days for a total of seven doses. On day 23, mice were euthanized, tumors were excised and weighed, and Ki67 expression levels were analyzed by immunohistochemistry.

### Immunohistochemistry (IHC)

Four-micrometer-thick paraffin-embedded sections from 6RK73-treated subcutaneous tumors were used for IHC to detect Ki67 protein expression. Antigen retrieval was performed in 10 mM citrate buffer at 100°C for 2 minutes. After cooling to room temperature, non-specific antigen binding and endogenous peroxidase activity were blocked using a peroxidase blocking reagent. Sections were incubated overnight at 4°C with a goat anti-human Ki67 antibody (1:100, Abcam, MA, USA). The next day, sections were incubated with a secondary antibody, followed by streptavidin-conjugated horseradish peroxidase (HRP) (Maixin Inc, China). Signals were visualized using DAB staining and analyzed under a bright-field microscope.

### Western blot analysis

Cells were lysed in RIPA buffer containing 1% phosphatase inhibitor and 1% protease inhibitor (Fdbio Science, Hangzhou, China). Protein concentrations were determined using a BCA Protein Assay Kit (Epizyme BioTech, Shanghai, China). Equal amounts of protein were separated via 10% SDS-PAGE and transferred onto PVDF membranes (Millipore, Danvers, MA, USA). Membranes were blocked with 5% bovine serum albumin (BSA, BioFroxx, Germany) and incubated overnight at 4°C with the following primary antibodies: Sp1 (21962-1-AP, Proteintech, 1:2000), c-Myc(10828-1-AP, Proteintech, 1:2000), AKT1 (10176-2-AP, Proteintech, 1:2000), P-AKT (80462-1-RR, Proteintech, 1:2000), USP7 (66514-1-Ig, Proteintech, 1:5000), GAPDH (AP0063, Bioworld, 1:10000), β-tubulin (AP0064, Bioworld, 1:5000), and Ubiquitin (10201-2-AP, Proteintech, 1:1000). After incubation with HRP-conjugated secondary antibodies for 1 hour at room temperature, signals were detected using the MiniChemiTM Chemiluminescence Imaging System (Sagecreation, Beijing, China).

### Cycloheximide chase and ubiquitination assays

Cells were treated with 50 μg/mL cycloheximide (CHX) (Selleck, Shanghai, China) for various time points. Protein lysates were prepared using RIPA buffer and SDS loading buffer, followed by boiling for 10 minutes. For ubiquitination assays, cells were pre-treated with 20 µM MG132 (Selleck) for 6 hours before protein extraction and immunoblotting.

### Co-immunoprecipitation (Co-IP)

Co-IP assays were performed using the Pierce Co-IP Kit (Thermo Scientific, MA, USA) following the manufacturer's instructions. Total protein was extracted, and samples were incubated overnight at 4°C with either specific antibodies or control IgG antibodies. The next day, protein A/G magnetic beads (Bimake, Shanghai, China) were added and incubated for 1 hour at room temperature with gentle agitation. After washing five times with buffer, bound proteins were eluted using RIPA buffer mixed with SDS (4:1) and boiled for 10 minutes.

### Statistical analysis

All statistical analyses were performed using SPSS 23.0 (SPSS Inc., Chicago, IL, USA). Data are presented as mean ± standard deviation (SD). Student's t-test was used to compare differences between two groups. A p-value < 0.05 was considered statistically significant (*p < 0.05, **p < 0.01, ***p < 0.001).

## Results

### 6RK73 suppresses ovarian cancer cell proliferation and impedes cell cycle progression *in vitro*

To investigate the effect of compound 6RK73 (Figure 1A), we first determined its half-maximal inhibitory concentration (IC50) in ovarian cancer cells using the CCK-8 assay. The results showed that the IC50 values for the ovarian cancer cell lines OVCAR3 and SKOV-3 were 10.62 µM and 12.90 µM, respectively (Figure 1B). Next, we assessed the impact of 6RK73 on cell proliferation. As shown in Figure 1C, 6RK73 reduced the viability of OVCAR3 and SKOV-3 cells in a dose-dependent manner. In colony formation assays, both the number and size of colonies progressively decreased with increasing concentrations of 6RK73 (Figure 1D). Since reduced cell proliferation is often associated with cell cycle arrest, we performed flow cytometry to analyze cell cycle distribution. The results demonstrated that the proportion of cells in the G2/M phase gradually increased with higher concentrations of 6RK73 (Figure 1E). These findings indicate that 6RK73 exhibits potent cytotoxic effects against ovarian cancer cells.

**Figure 2:**
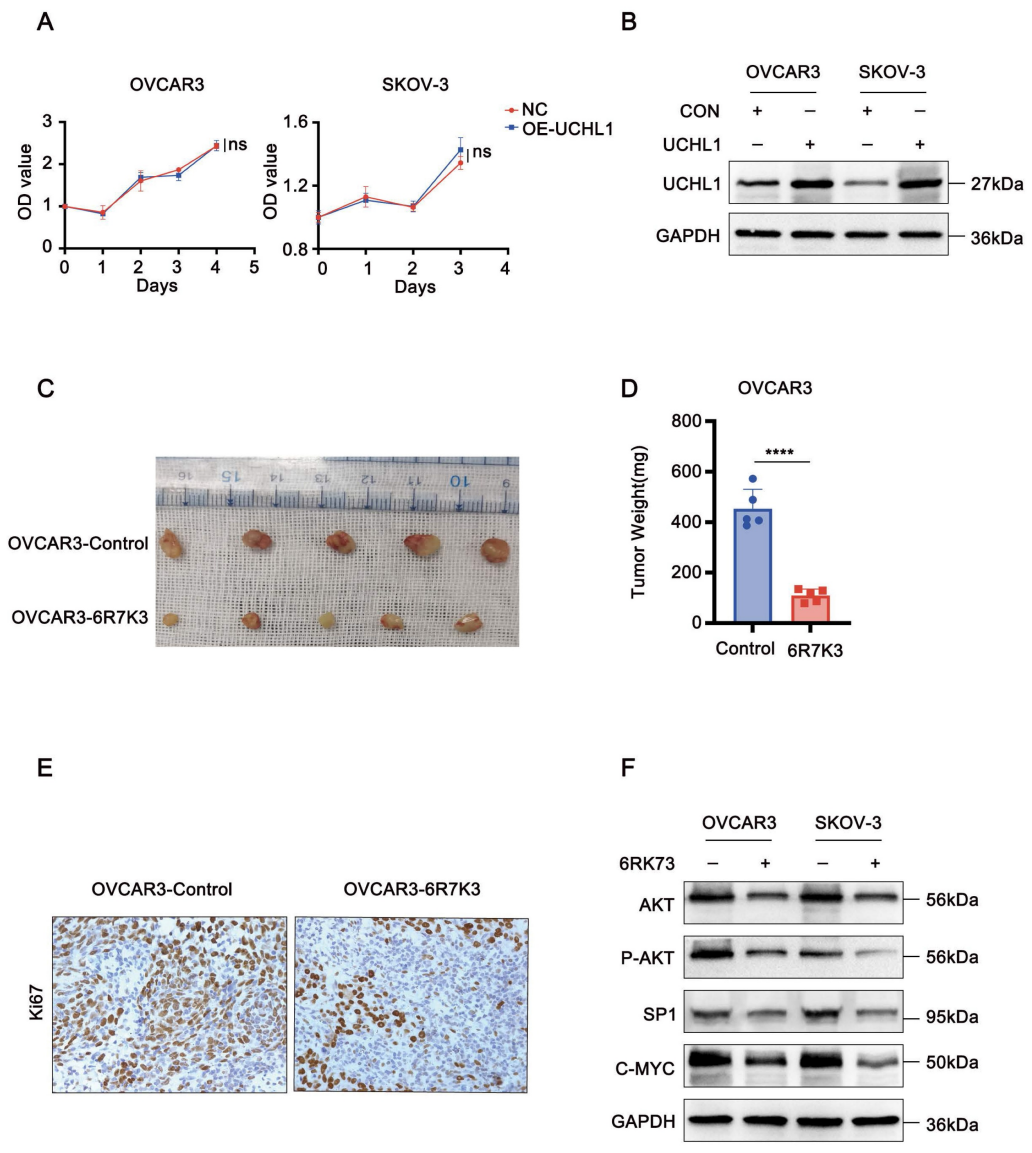
** 6RK73 suppresses the growth of ovarian cancer. (A-F)** A. Overexpression of UCHL1 does not affect the viability of OVCAR3 and SKOV-3 cells, as determined by the CCK-8 assay; B. The efficiency of UCHL1 overexpression was confirmed by Western blot analysis.; C and D. 6RK73 inhibits subcutaneous tumor growth in an ovarian cancer model; E. IHC staining indicates that Ki67 expression was lower in the 6RK73-treated group compared to the control group; F. Western blot analysis showing the effects of 6RK73 on AKT1, phosphorylated AKT1 (P-AKT1), c-Myc, and Sp1.

Ubiquitin C-Terminal Hydrolase L1 (UCHL1) is a thiol protease belonging to the C12 family of peptidases, which cleaves the peptide bond at the C-terminal glycine of ubiquitin. Previous studies have suggested that 6RK73 exerts antitumor effects by suppressing UCHL1 activity. However, UCHL1 has dual functions, and its role in ovarian cancer remains unclear. To determine whether the cytotoxicity of 6RK73 in ovarian cancer is linked to UCHL1 inhibition, we examined the function of UCHL1 in ovarian cancer cells. Unexpectedly, our results showed no statistically significant difference in cell proliferation between the UCHL1-overexpressing group and the control group (Figure 2A-B). Therefore, the cytotoxic effect of 6RK73 on OVCAR3 and SKOV-3 cells may be independent of UCHL1 activity.

### 6RK73 suppresses the tumor proliferation *in vivo*

To further evaluate the antitumor efficacy of 6RK73 *in vivo*, we established a subcutaneous ovarian cancer xenograft model in nude mice. Mice were treated with 10 mg/kg 6RK73 and tumor growth was monitored. The results demonstrated that 6RK73 significantly suppressed tumor growth, as indicated by reduced subcutaneous tumor size (Figure 2C-D). Furthermore, immunohistochemical staining revealed a marked decrease in Ki67 expression in the 6RK73-treated group compared to the control group (Figure 2E). These findings suggest that 6RK73 is a promising candidate for ovarian cancer treatment.

### 6RK73 inhibits the signaling pathway of AKT1/Sp1/c-Myc

AKT1 is a key oncogenic factor involved in the pathogenesis of various cancers, including lung, nasopharyngeal, breast, liver, and ovarian cancers^9^-^13^. Previous studies have demonstrated that Sp1 and c-Myc are major downstream effectors of AKT1 signaling^14^, ^15^. Western blot analysis of 6RK73-treated cells (5 µM) revealed a reduction in both total and phosphorylated AKT1 protein levels, accompanied by a significant downregulation of its downstream targets, Sp1 and c-Myc. These findings suggest that 6RK73 may inhibit tumor growth by inactivating the AKT1/Sp1/c-Myc signaling pathway (Figure 2F).

### Sp1 deubiquitinates C-Myc by binding c-Myc

**Figure 3:**
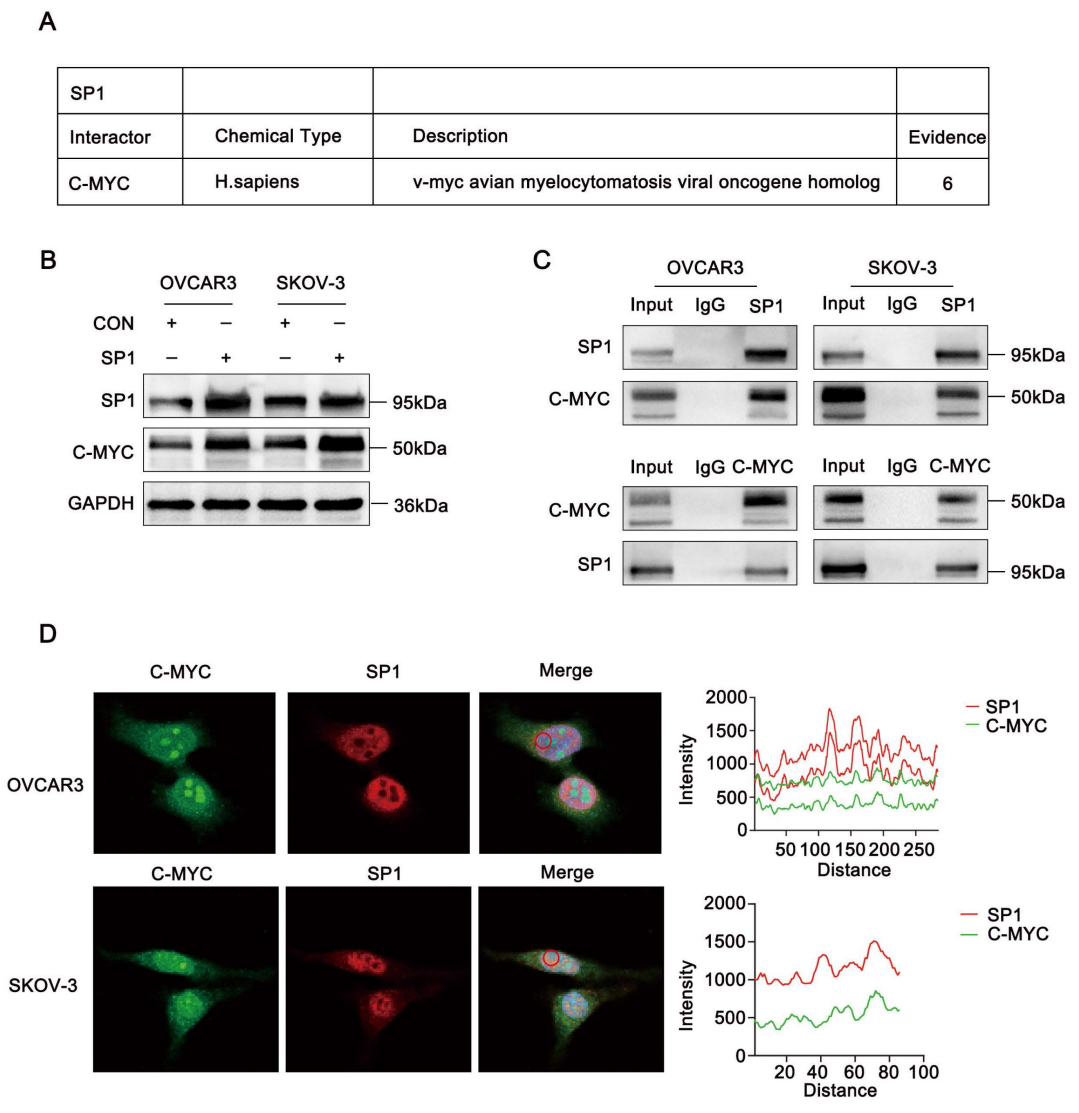
** Sp1 interacts with c-Myc. (A-D)** A. Sp1-interacting proteins as predicted by the BioGRID database; B. Changes in c-Myc protein expression in ovarian cancer cells transfected with the Sp1 plasmid; C. Co-IP analysis confirming the interaction between c-Myc and Sp1 in ovarian cancer cells; D. Immunofluorescence co-localization analysis demonstrating nuclear co-localization of Sp1 (Red) and c-Myc (Green).

Based on data from the BioGRID database, Sp1 was predicted to interact with c-Myc. (Figure 3A). These findings led us to hypothesize that Sp1 and c-Myc may regulate each other. As expected, overexpression of Sp1 in ovarian cancer cell lines resulted in a significant increase in c-Myc protein levels (Figure 3B). To confirm the interaction between Sp1 and c-Myc, we performed a Co-IP assay, which verified their association (Figure 3C). Additionally, immunofluorescence staining demonstrated that Sp1 and c-Myc predominantly colocalized in the cell nucleus (Figure 3D).

**Figure 4:**
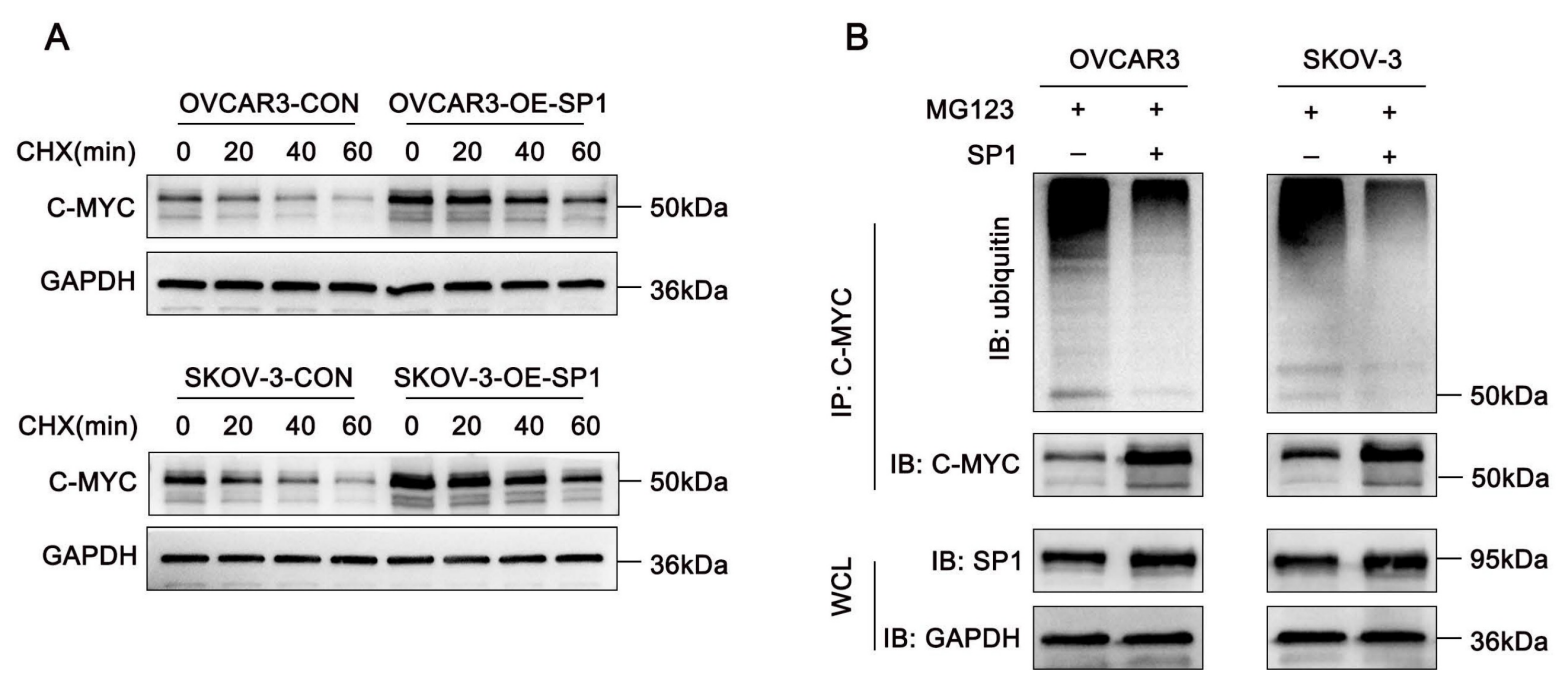
** Sp1 stabilizes c-Myc expression by reducing the level of c-Myc ubiquitination. (A-B)** A. Western blot analysis assessing the effects of CHX treatment on c-Myc stability in SP1-overexpressing and control cells; B. Effect of Sp1 overexpression on the ubiquitination level of c-Myc.

Since Sp1 interacts with c-Myc, we sought to determine how Sp1 regulates c-Myc protein expression. To investigate this, cycloheximide (CHX) chase assays were conducted in Sp1-overexpressing and control cells. The results indicated that Sp1 prolonged the half-life of c-Myc (Figure 4A). Furthermore, ubiquitination assays revealed that an increase in Sp1 levels was associated with a decreased ubiquitination of c-Myc (Figure 4B). These findings suggest that Sp1 stabilizes c-Myc expression by modulating its ubiquitination.

### USP7 deubiquitinates c-Myc and induces its expression

**Figure 5:**
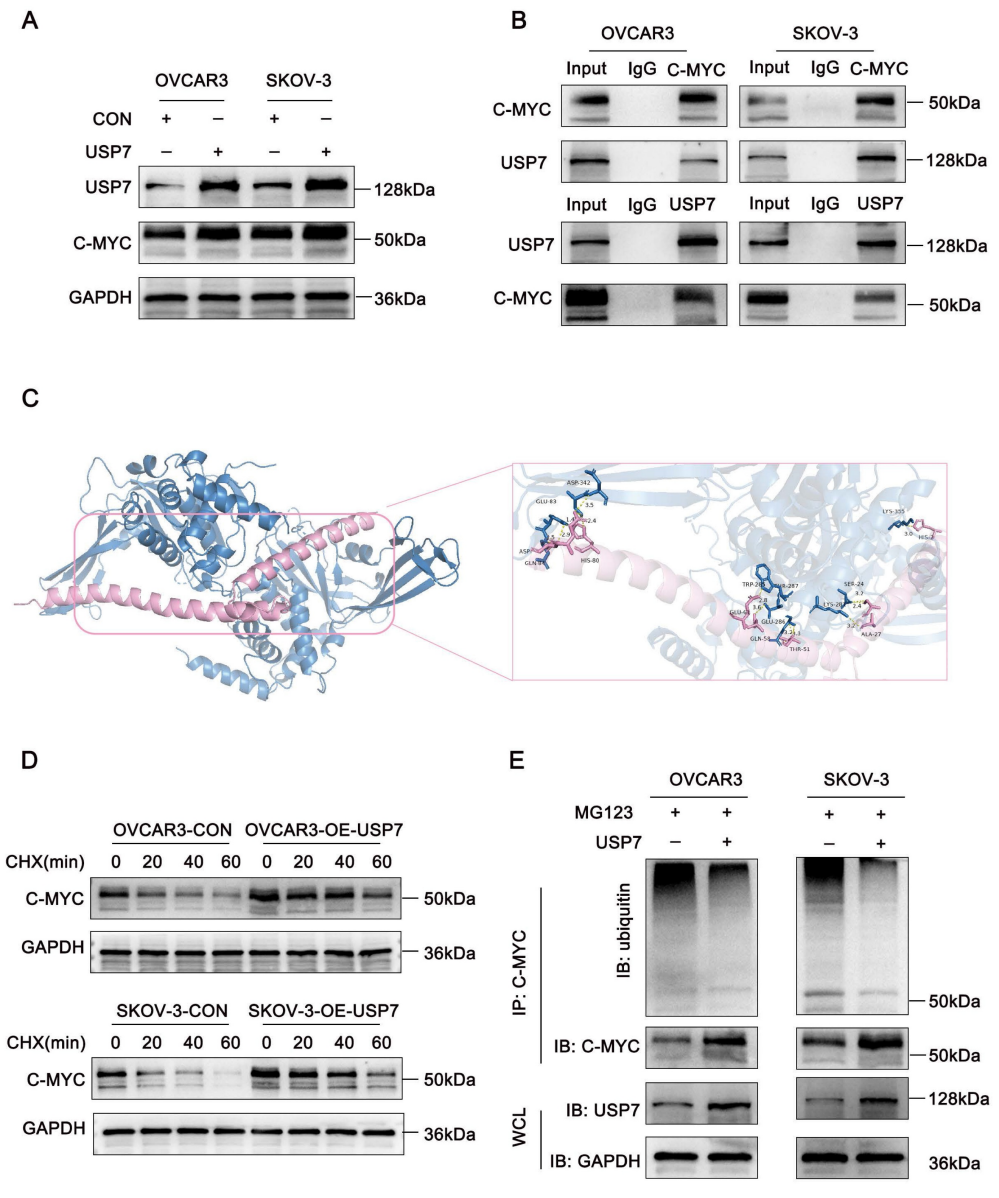
** USP7 binds to c-Myc and regulates its ubiquitination. (A-E).** A. Western blot analysis showing c-Myc expression levels following USP7 overexpression; B. Co-IP and Western blot assays confirming the interaction between c-Myc and USP7; C. Schematic representation of c-Myc and USP7 protein docking: c-Myc (purple) and USP7 (blue); D. Western blot analysis assessing the effects of CHX treatment on c-Myc stability in USP7-overexpressing and control cells; E. Co-IP and Western blot assays demonstrating the effect of USP7 overexpression on the ubiquitination level of c-Myc.

Intracellular proteins undergo ubiquitination through the ubiquitin ligase system, while deubiquitinating enzymes (DUBs) counteract this process by hydrolyzing ester, peptide, or isopeptide bonds at the carboxyl terminus of ubiquitin, thereby dynamically regulating protein degradation. Using the BioGRID database, we identified the deubiquitinating enzyme USP7 as a predicted interactor of both c-Myc and Sp1. To investigate whether USP7 regulates c-Myc expression, we performed western blot analysis, which confirmed that c-Myc protein levels were significantly increased in USP7-overexpressing cells (Figure 5A).

To further elucidate the molecular basis of the interaction between USP7 and c-Myc, we performed protein-protein docking to predict their potential binding sites and interaction modes. The crystal structures of USP7 (PDB ID: 1NK8) and c-Myc (PDB ID: 1NKP) were retrieved from the Protein Data Bank (PDB), and both protein structures were preprocessed by removing water molecules and bound ligands. Docking was carried out using the HDOCK server, and the top-ranked docking model was selected for structural analysis. Notably, the closest interaction was observed between residue Tyr-379 of USP7 and His-80 of c-Myc, forming a hydrogen bond with an interatomic distance of 1.4 Å (Fig. 5C). Furthermore, co-immunoprecipitation (Co-IP) assays confirmed the physical interaction between USP7 and c-Myc in ovarian cancer cells (Fig. 5B).

As a member of the peptidase C19 family, USP7 exhibits ubiquitin-hydrolyzing activity. To assess its functional impact on c-Myc stability, we examined c-Myc's half-life and ubiquitination levels. USP7 overexpression extended the half-life of c-Myc in ovarian cancer cells (Figure 5D). Moreover, c-Myc ubiquitination was significantly reduced upon USP7 overexpression (Figure 5E). Collectively, these findings suggest that USP7 binds to and regulates c-Myc in ovarian cancer.

### Sp1 recruits USP7 to deubiquitinate c-Myc

**Figure 6:**
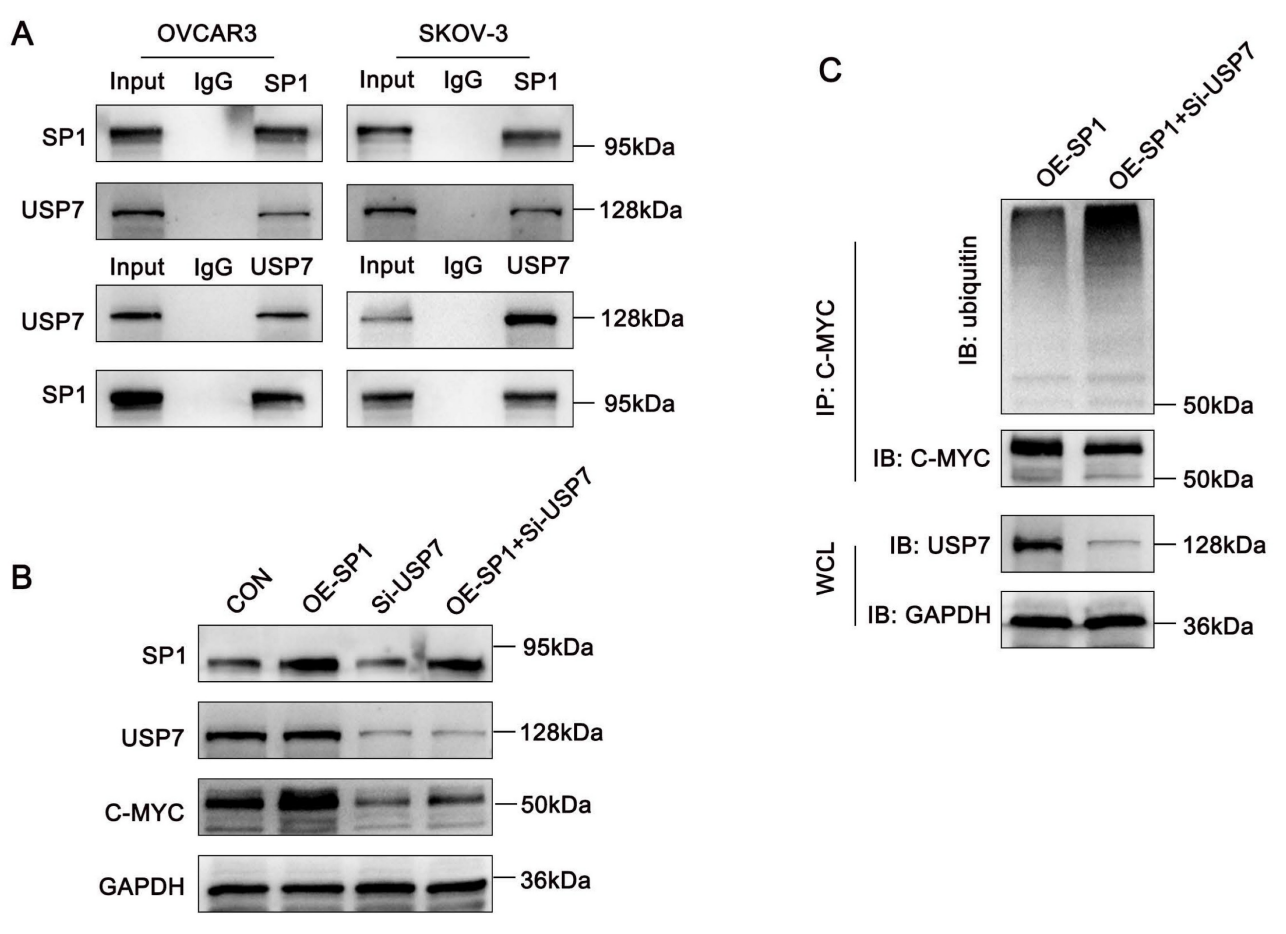
** Sp1 recruits USP7 to deubiquitinate C-MYC. (A-C).** A. Co-IP assays confirming the interaction between Sp1 and USP7; B. Western blot analysis detecting the effects of Sp1 overexpression, USP7 knockdown, and their co-transfection on c-Myc expression; C. Compared to Sp1 overexpression alone, co-transfection of the Sp1 overexpression plasmid and si-USP7 increased c-Myc ubiquitination levels.

Both Sp1 and USP7 interact with c-Myc and prevent its ubiquitin-mediated degradation. Thus, we hypothesized that Sp1 stabilizes c-Myc by recruiting USP7. To test this, we first performed Co-IP assays, which confirmed the interaction between Sp1 and USP7 (Figure 6A).

Next, to further investigate the regulatory relationship among Sp1, USP7 and c-Myc, we co-transfected ovarian cancer cells with a Sp1-overexpression plasmid and siRNA targeting USP7. Western blot analysis revealed that Sp1-induced upregulation of c-Myc was attenuated upon USP7 knockdown (Figure 6B). Furthermore, ubiquitination assays demonstrated that USP7 knockdown significantly increased c-Myc ubiquitination in Sp1-overexpressing ovarian cancer cells (Figure 6C). These results suggest that Sp1 stabilizes c-Myc by recruiting USP7 to deubiquitinate c-Myc.

### AKT1 reverses 6RK73-induced growth suppression in ovarian cancer

**Figure 7:**
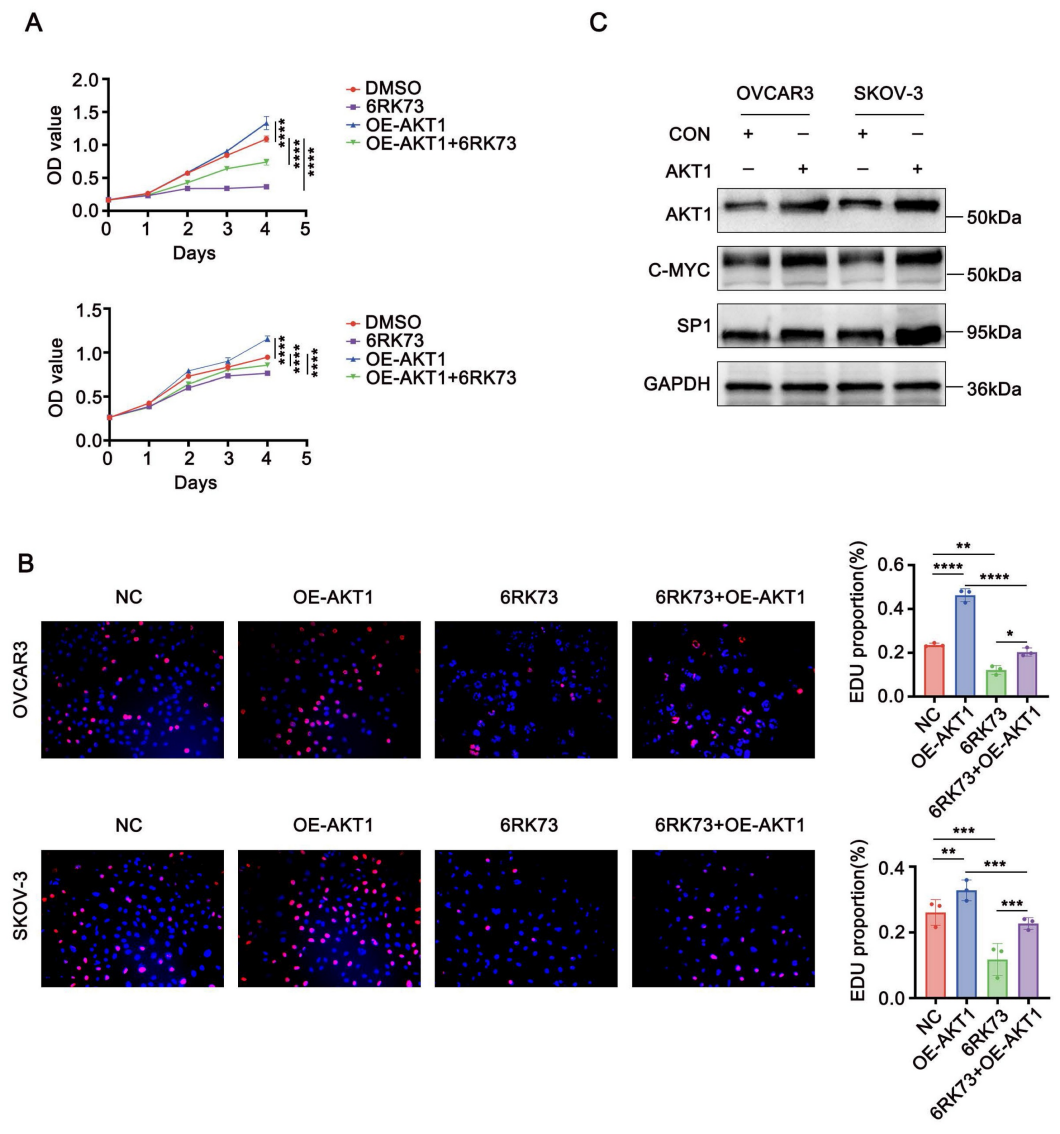
** AKT1 counteracts 6RK73-induced growth suppression in ovarian cancer. (A-C).** A. OVCAR3 and SKOV-3 cells, treated or untreated with 6RK73 and transfected or untransfected with the AKT1 plasmid, were subjected to CCK-8 assays to assess cell proliferation; B. EdU incorporation assays evaluating the effect of AKT1 transfection on 6RK73-induced cytotoxicity; C. Western blot analysis detecting Sp1 and c-Myc expression levels in AKT1-overexpressing and control cells.

To investigate whether AKT1 counteracts the suppressive effects of 6RK73, we overexpressed AKT1 in ovarian cancer cells following 6RK73 treatment. Cell proliferation and EdU incorporation assays demonstrated that AKT1 overexpression promoted cell proliferation and partially reversed the cytotoxic effects of 6RK73 in ovarian cancer cells (Figure 7A-B). Additionally, western blot analysis revealed that AKT1 overexpression significantly upregulated the expression of Sp1 and c-Myc (Figure 7C).

## Discussion

The development of ovarian cancer involves alterations in multiple signaling pathways and genes, including AKT1, WNT/β-catenin, mTOR, Notch, ERK/Ras, p53, and c-Myc^16^-^21^. Furthermore, recent studies indicate that the dysregulation of deubiquitinase genes, such as USP7 and USP1, contributes to ovarian cancer pathogenesis^22^, ^23^.

Ovarian cancer is a highly malignant tumor with limited therapeutic options. Current standard chemotherapy includes cisplatin, carboplatin, paclitaxel, and cyclophosphamide^24^-^27^. Additionally, targeted therapies such as Bevacizumab, Lukapoli, and PARP inhibitors play significant roles in ovarian cancer treatment^28^-^31^. However, the limited availability of effective therapeutic agents underscores the urgent need for novel anti-ovarian cancer drugs.

6RK73 is a newly developed inhibitor targeting the UCHL1 deubiquitinase. Preliminary studies have demonstrated its anticancer activity in breast cancer and renal cell carcinoma, but its potential efficacy in other malignancies, including ovarian cancer, remains unexplored. In this study, we first determined the IC50 values of 6RK73 in ovarian cancer cell lines OVCAR3 and SKOV3, which were 10.62 μM and 12.09 μM, respectively. To further evaluate its effects on ovarian cancer cell proliferation, we treated cells with increasing concentrations of 6RK73 and assessed cell viability using CCK-8 and colony formation assays. The results indicated that 6RK73 exerted dose-dependent inhibitory effects on ovarian cancer cell growth. Additionally, 6RK73 treatment arrested ovarian cancer cells at the G2/M transition. Finally, *in vivo* xenograft experiments demonstrated that 6RK73 suppressed subcutaneous tumor formation in nude mice, and Ki67 staining further supported its role as an anticancer agent.

UCHL1 is a member of the ubiquitin C-terminal hydrolases (UCHs) family of deubiquitinases. Emerging evidence suggests that UCHL1 is implicated in the pathogenesis of various cancers, including ovarian cancer^1^, ^4^, ^32^-^38^, where it exhibits dual roles as either a tumor promoter or suppressor. In breast cancer, urothelial bladder cancer, lung cancer, uterine serous carcinoma and glioma, UCHL1 functions as an oncogene. Conversely, in prostate cancer and nasopharyngeal carcinoma, UCHL1 acts as a tumor suppressor^4^, ^38^. However, its role in ovarian cancer remains ambiguous. Previous studies have demonstrated that UCHL1 is generally expressed at low levels in ovarian cancer, likely due to hypermethylation of its promoter region, thereby implicating it as a potential tumor suppressor gene^1^, ^33^. In our study, overexpression of UCHL1 in OVCAR3 and SKOV-3 ovarian cancer cell lines did not significantly affect cell proliferation, suggesting that UCHL1 may play a limited role in this context or that its tumor-suppressive function may require higher expression thresholds. Intriguingly, treatment with 6RK73 resulting in marked antiproliferative effects and was accompanied by downregulation of AKT1, Sp1, and c-Myc protein levels. This observation appears inconsistent with the proposed UCHL1-dependent mechanism of action. Therefore, we hypothesize that the cytotoxic effects of 6RK73 in ovarian cancer cells may occur through a UCHL1-independent mechanism, potentially involving suppression of the AKT1/Sp1/c-Myc signaling axis. AKT1 is a well-established oncogenic factor implicated in the development of multiple malignancies, including lung cancer, nasopharyngeal carcinoma, breast cancer, hepatocellular carcinoma, and ovarian cancer^9^-^13^. Among its downstream targets, Sp1 and c-Myc play critical roles in promoting tumorigenesis^14^, ^15^. Our study revealed that 6RK73 not only suppressed total and phosphorylated AKT1 protein levels but also significantly downregulated Sp1 and c-Myc expression, suggesting that 6RK73 exerts its growth-inhibitory effects by inactivating the AKT1/Sp1/c-Myc signaling axis.

Sp1 and c-Myc are well-known oncogenic transcription factors that drive tumor initiation and progression^39^-^43^. Notably, we observed that Sp1 physically interacts with c-Myc and co-localizes within the nucleus. Furthermore, Sp1 promotes c-Myc deubiquitination by recruiting the oncogenic deubiquitinase USP7, thereby stabilizing c-Myc protein and enhancing its expression in ovarian cancer^44^-^46^. These findings indicate that Sp1-mediated recruitment of USP7 contributes to c-Myc upregulation.

Finally, we transfected AKT1 into ovarian cancer cells with or without 6RK73 treatment and found that AKT1 overexpression reactivated AKT1/Sp1/c-Myc signaling and reversed 6RK73-induced growth suppression. Collectively, our findings suggest that 6RK73 is a promising anticancer agent that exerts cytotoxic and cell cycle-arresting effects by downregulating the AKT1/Sp1/c-Myc pathway, independent of UCHL1 inactivation.

## Supplementary Material

Supplementary figure.

## References

[1] Jin C, Yu W, Lou X, Zhou F, Han X, Zhao N (2013). UCHL1 Is a Putative Tumor Suppressor in Ovarian Cancer Cells and Contributes to Cisplatin Resistance. J Cancer.

[2] Liu S, González-Prieto R, Zhang M, Geurink PP, Kooij R, Iyengar PV (2020). Deubiquitinase Activity Profiling Identifies UCHL1 as a Candidate Oncoprotein That Promotes TGFβ-Induced Breast Cancer Metastasis. Clin Cancer Res.

[3] Mondal M, Conole D, Nautiyal J, Tate EW (2022). UCHL1 as a novel target in breast cancer: emerging insights from cell and chemical biology. Br J Cancer.

[4] Ummanni R, Jost E, Braig M, Lohmann F, Mundt F, Barett C (2011). Ubiquitin carboxyl-terminal hydrolase 1 (UCHL1) is a potential tumour suppressor in prostate cancer and is frequently silenced by promoter methylation. Mol Cancer.

[5] Bilguvar K, Tyagi NK, Ozkara C, Tuysuz B, Bakircioglu M, Choi M (2013). Recessive loss of function of the neuronal ubiquitin hydrolase UCHL1 leads to early-onset progressive neurodegeneration. Proc Natl Acad Sci U S A.

[6] Kooij R, Liu S, Sapmaz A, Xin BT, Janssen GMC, van Veelen PA (2020). Small-Molecule Activity-Based Probe for Monitoring Ubiquitin C-Terminal Hydrolase L1 (UCHL1) Activity in Live Cells and Zebrafish Embryos. J Am Chem Soc.

[7] Liu N, Chen Y, Yang L, Shi Q, Lu Y, Ma W (2022). Both SUMOylation and ubiquitination of TFE3 fusion protein regulated by androgen receptor are the potential target in the therapy of Xp11.2 translocation renal cell carcinoma. Clin Transl Med.

[8] Asangba AE, Chen J, Goergen KM, Larson MC, Oberg AL, Casarin J (2023). Diagnostic and prognostic potential of the microbiome in ovarian cancer treatment response. Sci Rep.

[9] Tian X, Wang R, Gu T, Ma F, Laster KV, Li X (2022). Costunolide is a dual inhibitor of MEK1 and AKT1/2 that overcomes osimertinib resistance in lung cancer. Mol Cancer.

[10] Yang L, Xiao L, Ma X, Tang M, Weng X, Chen X (2009). Effect of DNAzymes targeting Akt1 on cell proliferation and apoptosis in nasopharyngeal carcinoma. Cancer Biol Ther.

[11] George B, Gui B, Raguraman R, Paul AM, Nakshatri H, Pillai MR (2022). AKT1 Transcriptomic Landscape in Breast Cancer Cells. Cells.

[12] Yu M, Zeng M, Pan Z, Wu F, Guo L, He G (2020). Discovery of novel akt1 inhibitor induces autophagy associated death in hepatocellular carcinoma cells. Eur J Med Chem.

[13] Huang TT, Chiang CY, Nair JR, Wilson KM, Cheng K, Lee JM (2024). AKT1 interacts with DHX9 to Mitigate R Loop-Induced Replication Stress in Ovarian Cancer. Cancer Res.

[14] Priolo C, Pyne S, Rose J, Regan ER, Zadra G, Photopoulos C (2014). AKT1 and MYC induce distinctive metabolic fingerprints in human prostate cancer. Cancer Res.

[15] Choi JA, Jung YS, Kim JY, Kim HM, Lim IK (2016). Inhibition of breast cancer invasion by TIS21/BTG2/Pc3-Akt1-Sp1-Nox4 pathway targeting actin nucleators, mDia genes. Oncogene.

[16] Ediriweera MK, Tennekoon KH, Samarakoon SR (2019). Role of the PI3K/AKT/mTOR signaling pathway in ovarian cancer: Biological and therapeutic significance. Semin Cancer Biol.

[17] Belur Nagaraj A, Knarr M, Sekhar S, Connor RS, Joseph P, Kovalenko O (2021). The miR-181a-SFRP4 Axis Regulates Wnt Activation to Drive Stemness and Platinum Resistance in Ovarian Cancer. Cancer Res.

[18] Chen B, Jiang K, Wang H, Miao L, Lin X, Chen Q (2023). NOTCH Pathway Genes in Ovarian Cancer: Clinical Significance and Associations with Immune Cell Infiltration. Front Biosci (Landmark Ed).

[19] Hendrikse CSE, Theelen PMM, van der Ploeg P, Westgeest HM, Boere IA, Thijs AMJ (2023). The potential of RAS/RAF/MEK/ERK (MAPK) signaling pathway inhibitors in ovarian cancer: A systematic review and meta-analysis. Gynecol Oncol.

[20] Ramraj SK, Elayapillai SP, Pelikan RC, Zhao YD, Isingizwe ZR, Kennedy AL (2020). Novel ovarian cancer maintenance therapy targeted at mortalin and mutant p53. Int J Cancer.

[21] Jin Y, Qiu J, Lu X, Li G (2022). C-MYC Inhibited Ferroptosis and Promoted Immune Evasion in Ovarian Cancer Cells through NCOA4 Mediated Ferritin Autophagy. Cells.

[22] Zhu X, Zhang Y, Luo Q, Wu X, Huang F, Shu T (2021). The deubiquitinase USP11 promotes ovarian cancer chemoresistance by stabilizing BIP. Signal Transduct Target Ther.

[23] Wang Y, Luo X, Wu N, Liao Q, Wang J (2023). USP7 mediates TRAF4 deubiquitination to facilitate the malignant phenotype of ovarian cancer via the RSK4/PI3K/AKT axis. J Cancer Res Ther.

[24] Li J, Qi F, Su H, Zhang C, Zhang Q, Chen Y (2022). GRP75-faciliated Mitochondria-associated ER Membrane (MAM) Integrity controls Cisplatin-resistance in Ovarian Cancer Patients. Int J Biol Sci.

[25] Ngoi NY, Syn NL, Goh RM, Goh BC, Huang RY, Soon YY (2022). Weekly versus tri-weekly paclitaxel with carboplatin for first-line treatment in women with epithelial ovarian cancer. Cochrane Database Syst Rev.

[26] Tossetta G (2022). Metformin Improves Ovarian Cancer Sensitivity to Paclitaxel and Platinum-Based Drugs: A Review of In Vitro Findings. Int J Mol Sci.

[27] Zsiros E, Lynam S, Attwood KM, Wang C, Chilakapati S, Gomez EC (2021). Efficacy and Safety of Pembrolizumab in Combination with Bevacizumab and Oral Metronomic Cyclophosphamide in the Treatment of Recurrent Ovarian Cancer: A Phase 2 Nonrandomized Clinical Trial. JAMA Oncol.

[28] Cortez AJ, Tudrej P, Kujawa KA, Lisowska KM (2018). Advances in ovarian cancer therapy. Cancer Chemother Pharmacol.

[29] O'Malley DM (2019). New Therapies for Ovarian Cancer. J Natl Compr Canc Netw.

[30] Ogundipe OD, Olajubutu O, Adesina SK (2023). Targeted drug conjugate systems for ovarian cancer chemotherapy. Biomed Pharmacother.

[31] Moore KN, Oza AM, Colombo N, Oaknin A, Scambia G, Lorusso D (2021). Phase III, randomized trial of mirvetuximab soravtansine versus chemotherapy in patients with platinum-resistant ovarian cancer: primary analysis of FORWARD I. Ann Oncol.

[32] Jia Q, Wang H, Xiao X, Sun Y, Tan X, Chai J (2023). UCHL1 acts as a prognostic factor and promotes cancer stemness in cervical squamous cell carcinoma. Pathol Res Pract.

[33] Tangri A, Lighty K, Loganathan J, Mesmar F, Podicheti R, Zhang C (2021). Deubiquitinase UCHL1 Maintains Protein Homeostasis through the PSMA7-APEH-Proteasome Axis in High-grade Serous Ovarian Carcinoma. Mol Cancer Res.

[34] Zheng Y, Shi D, Chen L, Yang Y, Yao M (2023). UCHL1-PKM2 axis dysregulation is associated with promoted proliferation and invasiveness of urothelial bladder cancer cells. Aging (Albany NY).

[35] Ding X, Gu Y, Jin M, Guo X, Xue S, Tan C (2020). The deubiquitinating enzyme UCHL1 promotes resistance to pemetrexed in non-small cell lung cancer by upregulating thymidylate synthase. Theranostics.

[36] Kwan SY, Au-Yeung CL, Yeung TL, Rynne-Vidal A, Wong KK, Risinger JI (2020). Ubiquitin Carboxyl-Terminal Hydrolase L1 (UCHL1) Promotes Uterine Serous Cancer Cell Proliferation and Cell Cycle Progression. Cancers (Basel).

[37] Sanchez-Diaz PC, Chang JC, Moses ES, Dao T, Chen Y, Hung JY (2017). Ubiquitin carboxyl-terminal esterase L1 (UCHL1) is associated with stem-like cancer cell functions in pediatric high-grade glioma. PLoS One.

[38] Li L, Tao Q, Jin H, van Hasselt A, Poon FF, Wang X (2010). The tumor suppressor UCHL1 forms a complex with p53/MDM2/ARF to promote p53 signaling and is frequently silenced in nasopharyngeal carcinoma. Clin Cancer Res.

[39] Dhanasekaran R, Deutzmann A, Mahauad-Fernandez WD, Hansen AS, Gouw AM, Felsher DW (2022). The MYC oncogene - the grand orchestrator of cancer growth and immune evasion. Nat Rev Clin Oncol.

[40] Fatma H, Maurya SK, Siddique HR (2022). Epigenetic modifications of c-MYC: Role in cancer cell reprogramming, progression and chemoresistance. Semin Cancer Biol.

[41] Gao FY, Li XT, Xu K, Wang RT, Guan XX (2023). c-MYC mediates the crosstalk between breast cancer cells and tumor microenvironment. Cell Commun Signal.

[42] Xu X, Wang X, Chen Q, Zheng A, Li D, Meng Z (2023). Sp1 promotes tumour progression by remodelling the mitochondrial network in cervical cancer. J Transl Med.

[43] Dong X, Liu Z, Zhang E, Zhang P, Wang Y, Hang J (2021). USP39 promotes tumorigenesis by stabilizing and deubiquitinating SP1 protein in hepatocellular carcinoma. Cell Signal.

[44] Hou R, Li Y, Luo X, Zhang W, Yang H, Zhang Y (2022). ENKUR expression induced by chemically synthesized cinobufotalin suppresses malignant activities of hepatocellular carcinoma by modulating β-catenin/c-Jun/MYH9/USP7/c-Myc axis. Int J Biol Sci.

[45] Dai X, Lu L, Deng S, Meng J, Wan C, Huang J (2020). USP7 targeting modulates anti-tumor immune response by reprogramming Tumor-associated Macrophages in Lung Cancer. Theranostics.

[46] Liu JH, Yang HL, Deng ST, Hu Z, Chen WF, Yan WW (2022). The small molecule chemical compound cinobufotalin attenuates resistance to DDP by inducing ENKUR expression to suppress MYH9-mediated c-Myc deubiquitination in lung adenocarcinoma. Acta Pharmacol Sin.

