# Supplementary material for: Potential Anti-Cancer Drug 6RK73 Suppresses Ovarian Cancer Growth by Inactivating the AKT1/Sp1 Induced c-Myc Signaling Pathway
Source: J Cancer. 2026 Jan 1;17(2):245–56. doi: 10.7150/jca.113511 (PMC12825427; doi:10.7150/jca.113511)
Supplement: Supplementary file 1 — Supplementary figure. [file jcav17p0245s1.pdf]

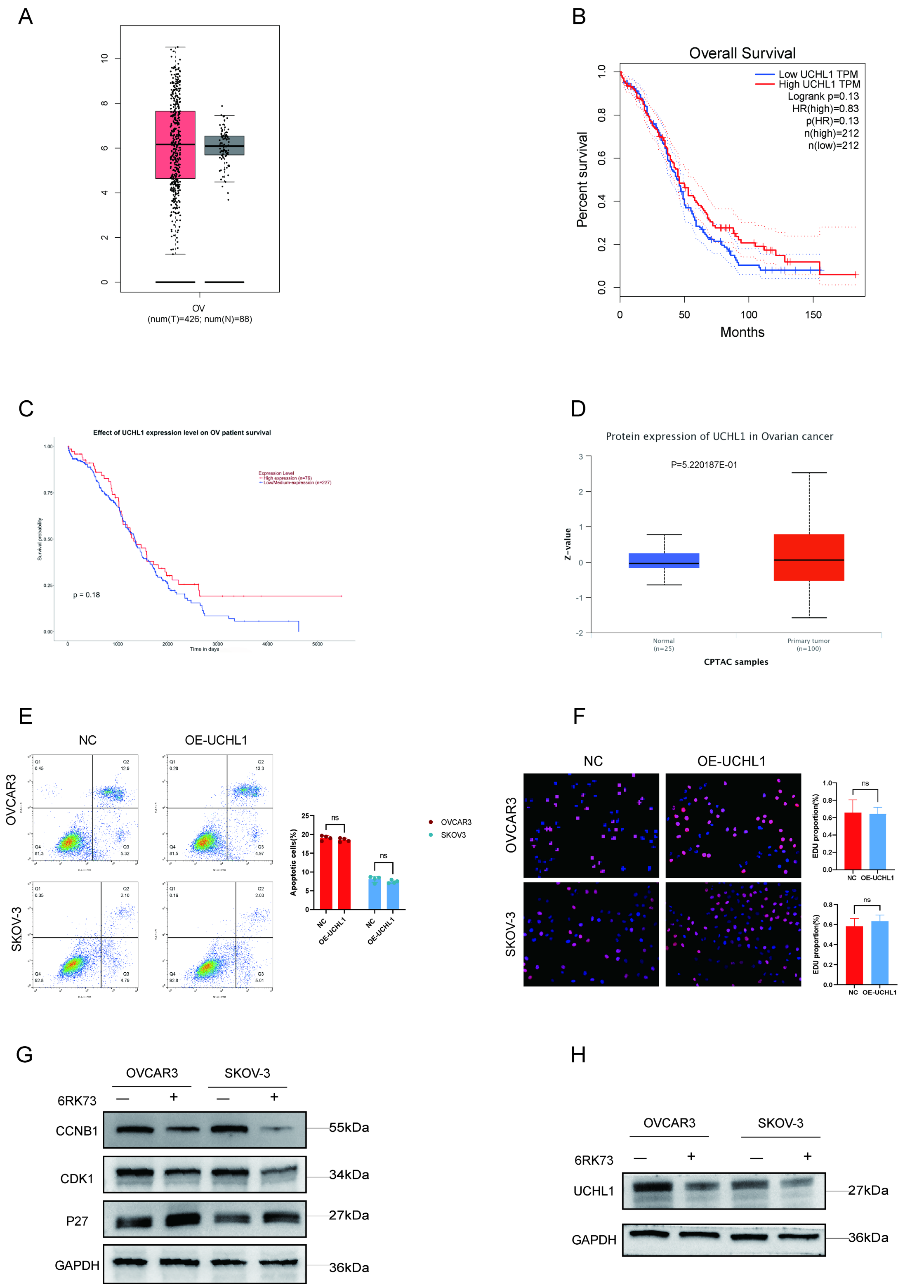

A: According to the GEPIA database analysis, there was no statistically significant difference in UCHL1 mRNA expression between normal ovarian tissues and ovarian cancer tissues (data without annotation indicate non-significance); B: Survival

analysis based on the GEPIA database showed no significant difference in overall survival between ovarian cancer patients with high versus low UCHL1 expression ($P > 0.05$); C: Survival analysis from the UALCAN database revealed no significant association between UCHL1 expression levels and overall survival in ovarian cancer patients ($P > 0.05$); D: UALCAN database analysis indicated that the difference in UCHL1 protein expression between normal ovarian tissues and ovarian cancer tissues was not statistically significant ($P > 0.05$); E: Flow cytometry analysis demonstrated no significant change in the proportion of apoptotic cells in OVCAR3 and SKOV-3 cells transfected with the UCHL1 plasmid compared to the control group; F: EDU assays showed no significant alteration in the proportion of proliferating cells following UCHL1 overexpression in OVCAR3 and SKOV-3 cells; G: Western blot analysis revealed that treatment with 6RK73 (5 μM) led to the downregulation of CCNB1 and CDK1, while upregulating the expression of P27 in OVCAR3 and SKOV-3 cells; H: Western blot analysis confirmed that UCHL1 protein levels were reduced in OVCAR3 and SKOV-3 cells following treatment with 6RK73 (5 μM).
